# Is Ultrasound an Accurate Alternative for Mammography in Breast Cancer Screening in an Asian Population? A Meta-Analysis

**DOI:** 10.3390/diagnostics10110985

**Published:** 2020-11-21

**Authors:** Jing Wang, Senshuang Zheng, Lanjun Ding, Xuan Liang, Yuan Wang, Marcel J.W. Greuter, Geertruida H. de Bock, Wenli Lu

**Affiliations:** 1 Department of Epidemiology, University of Groningen, University Medical Center Groningen, 9713AM Groningen, The Netherlands; j.wang@umcg.nl (J.W.); s.zheng02@umcg.nl (S.Z.); g.h.de.bock@umcg.nl (G.H.d.B.); 2 Department of Epidemiology and Health Statistics, School of Public Health, Tianjin Medical University, 22 Qixiangtai Street, Heping District, Tianjin 300070, China; 463254418ding@gmail.com (L.D.); lx199209@outlook.com (X.L.); wangyuan@tmu.edu.cn (Y.W.); 3 Collaborative Innovation Center of Chronic Disease Prevention and Control, Tianjin Medical University, 22 Qixiangtai Street, Heping District, Tianjin 300070, China; 4 Department of Radiology, University of Groningen, University Medical Center Groningen, 9713AM Groningen, The Netherlands; m.j.w.greuter@umcg.nl; 5 Department of Robotics and Mechatronics, University of Twente, 7522 NH Enschede, The Netherlands

**Keywords:** breast neoplasms, mass screening, meta-analysis, mammography, ultrasonography

## Abstract

In Asian countries, ultrasound has been proposed as a possible alternative for mammography in breast cancer screening because of its superiority in dense breasts, accessibility and low costs. This research aimed to meta-analyze the evidence for the diagnostic performance of ultrasound compared to mammography for breast cancer screening in Asian women. PubMed, Web of Science, and China National Knowledge Infrastructure databases were searched for studies that concurrently compared mammography and ultrasound in 2000–2019. Data extraction and risk of bias were performed according to the Preferred Reporting Items for Systematic Review and Meta-Analysis Protocols (PRISMA) statement. The primary outcome was the sensitivity and specificity. Bivariate random models were used to generate pooled estimates of diagnostic parameters and 95% confidence intervals (95% CI). In total, 4424 studies were identified of which six studies met the inclusion criteria with a sample size of 124,425 women. The pooled mean prevalence of the included studies was 3.7‰ (range: 1.2–5.7‰). The pooled sensitivity of mammography was significantly higher than that of ultrasound (0.81 [95% CI 0.71–0.88] versus 0.65 [95% CI 0.58–0.72], *p* = 0.03), but no significant differences were found in specificity (0.98 [95% CI: 0.94–1.00] versus 0.99 [95% CI: 0.97–1.00], *p* = 0.65). In conclusion, based on the currently available data on sensitivity alone, there is no indication that ultrasound can replace mammography in breast cancer screening in Asian women.

## 1. Introduction

Breast cancer is nowadays the main cause of cancer-related death in Asian women, with a dramatic increase in age-standardized incidence over recent years [1,2]. Data from China, South Korea, and Singapore showed that, during the period from 1988 to 2009, the estimated annual increase of the age-standardized incidence ranged from 2.0% to 6.4% [3]. In 2012, more than 600,000 new breast cancer cases were reported in Asia, accounting for 39% of all diagnosed breast cancers worldwide [2].

Currently, there is no universally effective method to lower the incidence of breast cancer [4,5]. However, regular screening could reduce disease burden as more cancers could be detected at earlier stages, allowing for better treatment and survival [6]. Although controversies about the efficiency of breast cancer screening still exist, several successful organized screening programs introduced in European and North American countries have shown that breast cancer mortality can be reduced by 20% by regular mammography screening for women aged 50 to 69 years [7,8,9].

Several Asian countries, such as Japan, Korea, and Singapore, have also organized breast cancer screening programs using mammography as the main screening modality [10,11,12]. However, whether the typical mammography-based screening programs in western countries could also be efficient in an Asian population in terms of early diagnosis, mortality reduction, and cost-effectiveness is still unclear. Asian women tend to have smaller and more dense breasts compared to Western women, and the mean onset age of breast cancer for Asian women is around 40–50 years, which is 10 years younger than that for Western women [13,14,15]. Moreover, the sensitivity of mammography correlates negatively with breast density and is especially limited in younger women [16]. In addition, the costs and accessibility of mammography are important issues for many Asian countries with relatively limited medical resources [2,17]. Therefore, mammography-based screening in Asian women may not be as efficient as in Western women, and ultrasound has been proposed as a possible, more favorable alternative [2].

Compared with mammography, ultrasound is a cheaper, radiation-free, and less strenuous modality. Studies have shown that adjunctive ultrasound could detect additional cancers in women with negative mammograms and that it typically outperforms mammography in women with dense breast tissue [18,19]. However, whether ultrasound could serve as an accurate modality for breast cancer screening in Asian women is unclear. Therefore, we aimed to compare the diagnostic performance of ultrasound with mammography as a breast cancer screening modality in Asian women by a meta-analysis.

## 2. Materials and Methods

This systematic review and meta-analysis was performed according to the Preferred Reporting Items for Systematic Review and Meta-Analysis Protocols (PRISMA) statement [20] and the Cochrane Handbook for Systematic Reviews of Diagnostic Test Accuracy (version 1.0.0, 2013) [21]. The PROSPERO registration number of this study was CRD42017070617.

### 2.1. Search Strategy and Selection Criteria

A literature search was conducted for studies published between 1 January 2000 and 31 December 2019. PubMed, Web of Science, and China National Knowledge Infrastructure databases were searched with the following keywords: “breast neoplasms”, “breast cancer”, “mass screening”, “ultrasound”, “radiography”, “mammography”, and “Asia”. Study titles, abstracts, and texts were screened independently by two authors (J.W. and L.D.). An example of the search strategy in PubMed can be found in Table S1. Disagreements between the two authors were resolved by consensus with a third author (W.L.). All searches were supplemented by checking the references of the identified articles.

### 2.2. Inclusion Criteria

Studies were included if they met the following criteria: (I) original study; (II) participants were Asian women who live in Asian countries and were not diagnosed with breast cancer before; (III) population-wide breast cancer screening; (IV) the study had a concurrent design that compared mammography and ultrasound at the same time; and (V) the diagnostic standard included biopsy with follow-up results.

### 2.3. Data Extraction

Data were extracted independently by two authors (J.W. and S.Z.) and listed in a standardized extraction table including: author names, year of publication, study design, country, starting age of screening, breast cancer risk, follow-up length, screening interval (years), sample size, publication type, program duration, participation rate, and the numbers of true positives, false positives, false negatives, and true negatives.

### 2.4. Quality Assessment

Two authors (J.W. and S.Z.) used the Revised Tool for Quality Assessment of Diagnostic Accuracy Studies (QUADAS-2) as recommended by the Cochrane Handbook for Systematic Reviews of Diagnostic Test Accuracy [22]. Disagreements on the risk of bias were resolved by group discussion with other authors. The risk of bias and concerns about the relevance of each study were rated as “low” or “high”; in addition, studies were rated as “unclear” if there was insufficient information to make a judgement.

### 2.5. Data Analysis

As heterogeneity was expected to be high in the meta-analysis of diagnostic test accuracy studies, a bivariate random model was used to estimate the pooled estimates of sensitivity, specificity, and diagnostic odds ratios (DORs) with 95% confidence intervals (95% CI) [23]. The DOR summarizes the diagnostic accuracy of the test as a single indicator that describes how many times higher the odds are of obtaining a test positive result in a diseased rather than a non-diseased person [23]. The value of a DOR ranges from 0 to infinity, with a higher value suggesting a better test performance [24]. Sensitivity was the main outcome of our study. To evaluate whether the pooled sensitivity and specificity of both modalities differed, the model was expanded by adding a modality-type covariate [25], and likelihood ratio tests were used to test significance. To assess the sensitivity and specificity of mammography and ultrasound separately, forest plots were used.

All analyses used two-sided tests and a significance level of 0.05. Statistical analyses were performed by Stata/SE 15.0 (StataCorp, College Station, TX, USA), and RevMan (Version 5.3 Copenhagen: The Nordic Cochrane Centre, The Cochrane Collaboration, 2014) was used to display the results graphically.

## 3. Results

### 3.1. Studies Characteristics

**Figure 1:**
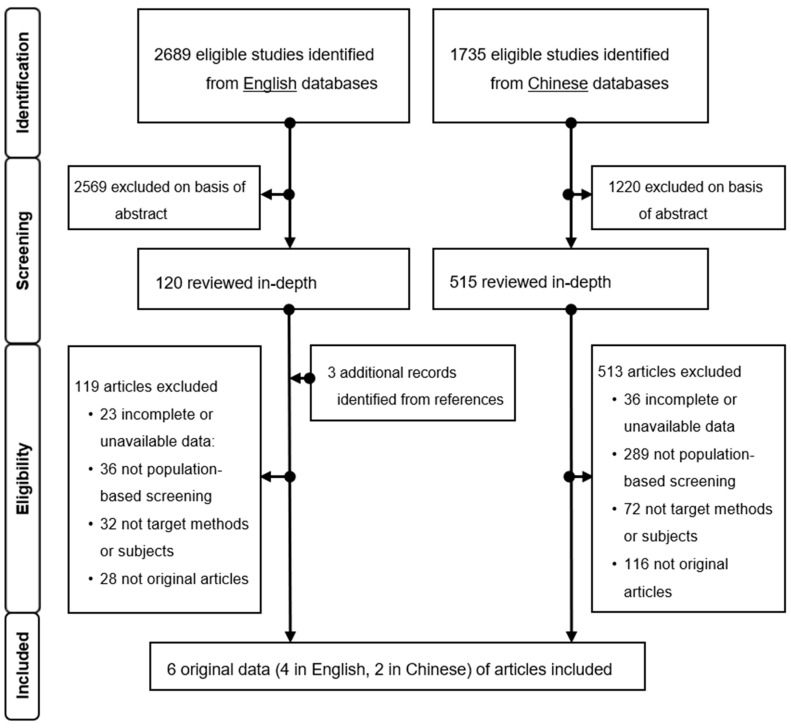
Flow chart of the literature search.

Our search returned 2689 English and 1735 Chinese records, six of which met the inclusion criteria (Figure 1). Table 1 details the characteristics of the included studies. We identified two multicenter randomized controlled trials (RCT) and four cohort studies (Table 1) [26,27,28,29,30,31]. The diagnostic accuracy data for the included studies is summarized in Table 2. The screening durations ranged from 24 to 87 months. Follow-up time varied among studies, ranging from 12 months to 24 months. Two studies used digital mammography [26,30]; two studies used screen-film mammography [27,29]; and the other two studies did not specify the type of mammography [28,31].

### 3.2. Quality Assessment

**Figure 2:**
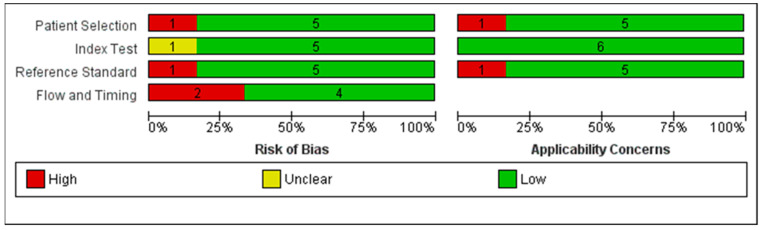
Risk of bias and applicability concerns: review authors’ judgements about each domain presented as a percentage across the included studies.

The results of the QUADAS-2 assessment are presented in Figure 2. Concerning participant selection in the six included studies, the risk of bias in participant selection was high in one study and low in the remaining five studies (Figure 2). In two studies, the risk of bias was high and was mainly related to the “Flow and Timing” domain.

### 3.3. Synthesis Results

**Figure 3:**
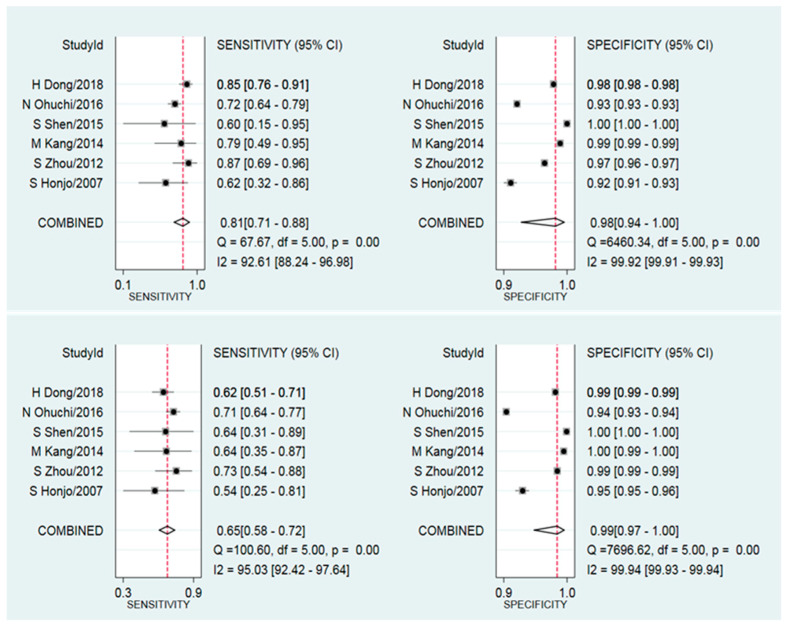
Forest plot of the sensitivity (**left**) and specificity (**right**) of mammography (**top**) and ultrasound (**bottom**) breast cancer screening in Asian women.

The pooled estimates of the mean sensitivity and specificity of mammography were 0.81 (95% CI: 0.71–0.88) and 0.98 (95% CI: 0.94–1.00), respectively (Figure 3), and of ultrasound were 0.65 (95% CI: 0.58–0.72) and 0.99 (95% CI: 0.97–1.00), respectively (Figure 3). The pooled DOR estimates for mammography and ultrasound were 253 (42–1541) and 179 (58–552), respectively. The likelihood ratio tests showed that mammography had a higher sensitivity (*p* = 0.03) but no difference (*p* = 0.65) in specificity compared to ultrasound. The potential source of heterogeneity and publication bias was not applicable to be explored because of limited studies.

## 4. Discussion

In this meta-analysis, we found that the pooled sensitivity of mammography was significantly higher than the pooled sensitivity of ultrasound in Asian women (0.81 [95% CI 0.71–0.88] versus 0.65 [95% CI 0.58–0.72], *p* = 0.03). However, no significant differences were found in specificity between both modalities. Although there was a high degree of heterogeneity in the included studies, potential sources of heterogeneity could not be explored because of limited data.

Ultrasound has been proposed as a possible alternative to mammography in Asian countries, which is supported by studies showing a trend that screening with ultrasound has a higher sensitivity compared with mammography in Asian women [32,33,34,35]. However, the evaluation of the design of these studies shows that these studies only evaluated one screening modality (either mammography or ultrasound). As such, these studies are more prone to bias, and it can be expected that these studies present more favorable results regarding the modality under study. In our study, we included only studies that performed a concurrent analysis of breast screening with ultrasound and mammography, and we did not find that mammography could be replaced by ultrasound in terms of sensitivity.

The main reason why ultrasound was proposed as a possible alternative to mammography in Asian countries is its superior performance in younger women and women with dense breasts. Currently, there are debates about differences in peak onset age and breast density in Asian and Western women [13]. Though some studies have shown that the peak onset age in Asian women is around 40–50 years, which is at least 10 years earlier than in Western women [36,37], other studies have argued that the earlier peak age in Asian women is a transient phenomenon during a westernizing process as the peak onset age in Japan, Korea, and urban China has increased and is now comparable to western countries [38,39,40]. In addition, there are conflicting reports about ethnicity differences in breast density. Rajaram et al., for example, found that mammographic density was significantly higher in Asian women than in Caucasian women [15]. By contrast, several studies showed that higher breast density in Asian women is associated with breast size and body mass index rather than ethnicity differences [14,41].

In some Asian countries, such as Japan and China, ultrasound has been recommended as an adjunct modality to mammography in breast cancer screening [26,42]. Several studies have shown that ultrasound could improve cancer detection in women at a younger age and in women with dense breasts and negative mammogram results [26,43]. For example, the Japan Strategic Anti-cancer Randomized Trial (J-START), one of the largest randomized clinical trials that used adjunctive ultrasound as a screening modality in Asian women, showed that mammography plus ultrasound had a higher sensitivity and a higher cancer detection rate compared to mammography alone in women aged 40–49 (sensitivity: 91% vs. 77%; cancer detection rate: 0.50% vs. 0.32%, respectively) [26]. In addition, a meta-analysis focusing on women with dense breasts and negative mammography results showed that adjunctive ultrasound screening resulted in a 40% average increase of the cancer detection rate [43]. However, whether ultrasound improves cancer detection remains controversial. A recent retrospective analysis of the Breast Cancer Surveillance Consortium (BCSC) showed that, for the general population, the cancer detection rates were similar for mammography plus ultrasound and mammography alone (5.4 vs. 5.5 per 1000 screens, respectively), albeit with a higher false-positive rate in the mammography plus ultrasound group (55 vs. 22 per 1000, respectively) [44].

The estimation of sensitivity and specificity can be influenced by population characteristics and study design. Based on our analysis, the pooled sensitivity of mammography in the Asian population was 81%, which was relatively lower than 87% in a US population [45]. In most of the included studies, the starting age of screening was around 30–35 years, which was much younger than in other breast screening programs [7,8,9]. This can result in lower estimations for both sensitivity and specificity [16,34,46]. Secondly, most of the included studies were screening trials in which only the results of the first round were reported. This can result in an overestimation of sensitivity as prevalent cancers could also be detected by screening in the first round [47]. Thirdly, almost all studies applied a short follow-up time of one year, except the study of Ohuchi et al. [26], which might lead to a higher pooled specificity as the number of true negatives was overestimated [48]. In this analysis, although the sensitivity of ultrasound is relatively low (65%), the pooled specificity of ultrasound reached an unrealistically high value of 99%. We considered that the short follow-up time might be one of the possible reasons for such a high specificity.

There are some limitations of this study. Firstly, as only a few studies reported data regarding interval cancers and as varied screening intervals were applied, we were not able to perform a quantitative analysis on interval cancers. Secondly, the risk of bias was highest in the flow and timing domain, primarily because of reduced participation and loss to follow-up, and the pooled sensitivity estimates may, therefore, have been slightly overestimated. Thirdly, there are only a few screening programs that use ultrasound as the routine screening method, and only six studies could be included, with four from China. Thus, the results for ultrasound need careful consideration when extrapolating to other Asian countries. Lastly, although the heterogeneity was expected to be high in this type of meta-analysis, there were too few studies included to perform adequate subgroup analyses, and, therefore, potential sources of heterogeneity could not be explored.

## 5. Conclusions

Based on the current sensitivity data, there is no indication that ultrasound can replace mammography in breast cancer screening in Asian women. However, whether mammography screening should be recommended in Asian countries needs careful consideration. Future research on the long-term outcome is required and should be accompanied by an assessment of the cost-effectiveness of mammography screening in Asian settings.

## Figures and Tables

**Table 1:** Characteristics of the included breast cancer screening studies.

First Author [ref]	Year of Publication	Study Design	Country	Starting Age	Risk Level	Follow-Up (Year)
Ohuchi [26]	2016	RCT	Japan	40	High	2.0
Honjo [27]	2007	Cohort	Japan	30	Average	1.0
Zhou [28]	2012	Cohort	China	35	High	0.5–0.8
Kang [29]	2014	Cohort	China	35	Average	1.0
Shen [30]	2015	RCT	China	30	High	1.0
Dong [31]	2018	Cohort	China	45	Average	1.0

Abbreviations: RCT = Randomized clinical trial.

**Table 2:** Diagnostic accuracy of mammography and ultrasound in the included studies.

First Author [ref]	Method	Sample Size	Crude Prevalence‰ (95%CI)	TP (%)	FP (%)	FN (%)	TN (%)	PPV (%)	NPV (%)
Ohuchi [26]	US	36,049	5.6 (4.9–6.4)	143 (0.4)	2289 (6.3)	59 (0.2)	33,558 (93.1)	5.9	99.8
	MG	35,130	4.3 (3.7–5.1)	109 (0.3)	2576 (7.3)	43 (0.1)	32,402 (92.2)	4.1	99.9
Honjo [27]	US	3453	3.8 (2.0–6.4)	7 (0.2)	158 (4.6)	6 (0.2)	3282 (95.0)	4.2	99.8
	MG	3453	3.8 (2.0–6.4)	8 (0.2)	271 (7.8)	5 (0.2)	3169 (91.8)	2.9	99.8
Zhou [28]	US	7017	4.3 (2.9–6.1)	22 (0.3)	72 (1.0)	8 (0.1)	6915 (98.6)	23.4	99.9
	MG	7020	4.3 (2.9–6.1)	26 (0.4)	220 (3.1)	4 (0.1)	6770 (96.4)	10.6	99.9
Kang [29]	US	2471	5.7 (3.1–9.5)	9 (0.4)	9 (0.4)	5 (0.2)	2448 (99.0)	50.0	99.8
	MG	2471	5.7 (3.1–9.5)	11 (0.5)	24 (1.0)	3 (0.1)	2433 (98.4)	31.4	99.9
Shen [30]	US	4214	2.6 (1.3–4.7)	7 (0.2)	3 (0.1)	4 (0.1)	4200 (99.6)	70.0	99.9
	MG	4170	1.2 (0.4–2.8)	3 (0.1)	1 (0.0)	2 (0.1)	4164 (99.8)	75.0	99.9
Dong [31]	US	31,918	3.1 (2.5–3.8)	61 (0.2)	389 (1.2)	38 (0.1)	31,430 (98.5)	13.6	99.9
	MG	31,918	3.1 (2.5–3.8)	84 (0.3)	604 (1.9)	15 (0.1)	31,215 (97.7)	12.2	99.9

Abbreviations: MG = Mammography; US = Ultrasound; CI = Confidence interval; TP = True positive; FP = False negative; FN = False negative; TN = True negative; PPV = Positive predictive value; NPV = Negative predictive value.

## Supplementary Materials

The following are available online at https://www.mdpi.com/2075-4418/10/11/985/s1, Table S1. Search strategy in MEDLINE/PubMed.

## References

[1] DeSantis C.E., Bray F., Ferlay J., Lortet-Tieulent J., Anderson B.O., Jemal A. (2015). International Variation in Female Breast Cancer Incidence and Mortality Rates. Cancer Epidemiol. Biomark. Prev..

[2] Fan L., Goss P.E., Strasser-Weippl K.K. (2015). Current Status and Future Projections of Breast Cancer in Asia. Breast Care.

[3] Sung H., Rosenberg P.S., Chen W.-Q., Hartman M., Lim W.-Y., Chia K.S., Mang O.W.-K., Chiang C.-J., Kang D., Ngan R.K.-C. (2015). Female Breast Cancer Incidence Among Asian and Western Populations: More Similar Than Expected. J. Natl. Cancer Inst..

[4] Moss S.M., Wale C., A Smith R., Evans A., Cuckle H., Duffy S.W. (2015). Effect of mammographic screening from age 40 years on breast cancer mortality in the UK Age trial at 17 years’ follow-up: A randomised controlled trial. Lancet Oncol..

[5] Tabár L., Yen M.-F., Vitak B., Chen H.-H.T., Smith R.A., Duffy S.W. (2003). Mammography service screening and mortality in breast cancer patients: 20-year follow-up before and after introduction of screening. Lancet.

[6] De Munck L., Fracheboud J., De Bock G.H., Heeten G.J.D., Siesling S., Broeders M.J.M. (2018). Is the incidence of advanced-stage breast cancer affected by whether women attend a steady-state screening program?. Int. J. Cancer.

[7] Gøtzsche P.C., Jørgensen K.J. (2013). Screening for breast cancer with mammography. Cochrane Database Syst. Rev..

[8] Marmot M., Altman D., Cameron D., Dewar J., Thompson S., Wilcox M. (2013). The benefits and harms of breast cancer screening: An independent review. Br. J. Cancer.

[9] Humphrey L.L., Helfand M., Chan B.K., Woolf S.H. (2002). Breast Cancer Screening: A Summary of the Evidence for the U.S. Preventive Services Task Force. Ann. Intern. Med..

[10] Hamashima C., Hattori M., Honjo S., Kasahara Y., Katayama T., Nakai M., Nakayama T., Morita T., Ohta K., Japanese Research Group for the Development of Breast Cancer Screening Guidelines (2016). The Japanese Guidelines for Breast Cancer Screening. Jpn. J. Clin. Oncol..

[11] Lee E.H., Park B., Kim N.-S., Seo H.-J., Ko K.L., Min J., Shin M.-H., Lee K., Lee S., Choi N. (2015). The Korean guideline for breast cancer screening. J. Korean Med Assoc..

[12] Loy E.Y., Molinar D., Chow K.Y., Fock C. (2015). National Breast Cancer Screening Programme, Singapore: Evaluation of participation and performance indicators. J. Med Screen..

[13] Leong S.P.L., Shen Z.-Z., Liu T.-J., Agarwal G., Tajima T., Paik N.-S., Sandelin K., Derossis A., Cody H., Foulkes W.D. (2010). Is Breast Cancer the Same Disease in Asian and Western Countries?. World J. Surg..

[14] Maskarinec G., Meng L., Ursin G. (2001). Ethnic differences in mammographic densities. Int. J. Epidemiol..

[15] Rajaram N., Mariapun S., Eriksson M., Tapia J., Kwan P.Y., Ho W.K., Harun F., Rahmat K., Czene K., Taib N.A.M. (2017). Differences in mammographic density between Asian and Caucasian populations: A comparative analysis. Breast Cancer Res. Treat..

[16] Armstrong K., Moye E., Williams S., Berlin J.A., Reynolds E.E. (2007). Screening Mammography in Women 40 to 49 Years of Age: A Systematic Review for the American College of Physicians. Ann. Intern. Med..

[17] Rivera-Franco M.M., Leon-Rodriguez E. (2018). Delays in Breast Cancer Detection and Treatment in Developing Countries. Breast Cancer Basic Clin. Res..

[18] Nothacker M., Duda V., Hahn M., Warm M., Degenhardt F., Madjar H., Weinbrenner S., Albert U.-S. (2009). Early detection of breast cancer: Benefits and risks of supplemental breast ultrasound in asymptomatic women with mammographically dense breast tissue. A systematic review. BMC Cancer.

[19] Hwang J.-Y., Han B.-K., Ko E.Y., Shin J.H., Hahn S.Y., Nam M.Y. (2015). Screening Ultrasound in Women with Negative Mammography: Outcome Analysis. Yonsei Med. J..

[20] Moher D., Shamseer L., Clarke M., Ghersi D., Liberati A., Petticrew M., Shekelle P., Stewart L.A., PRISMA-P Group (2015). Preferred reporting items for systematic review and meta-analysis protocols (PRISMA-P) 2015 statement. Syst. Rev..

[21] Deeks J., Wisniewski S., Davenport C. (2013). Chapter 4: Guide to the contents of a Cochrane Diagnostic Test Accuracy Protocol. Cochrane Handbook for Systematic Reviews of Diagnostic Test Accuracy.

[22] Whiting P.F., Rutjes A.W., Westwood M.E., Mallett S., Deeks J.J., Reitsma J.B., Leeflang M.M., Sterne J.A., Bossuyt P.M. (2011). QUADAS-2: A Revised Tool for the Quality Assessment of Diagnostic Accuracy Studies. Ann. Intern. Med..

[23] Macaskill P., Gatsonis C., Deeks J., Harbord R., Takwoingi Y. (2010). Chapter 10: Analysing and Presenting Results. Cochrane Handbook for Systematic Reviews of Diagnostic Test Accuracy.

[24] Glas A.S., Lijmer J.G., Prins M.H., Bonsel G.J., Bossuyt P.M.M. (2003). The diagnostic odds ratio: A single indicator of test performance. J. Clin. Epidemiol..

[25] Leeflang M. (2014). Systematic reviews and meta-analyses of diagnostic test accuracy. Clin. Microbiol. Infect..

[26] Ohuchi N., Suzuki A., Sobue T., Kawai M., Yamamoto S., Zheng Y.-F., Shiono Y.N., Saito H., Kuriyama S., Tohno E. (2016). Sensitivity and specificity of mammography and adjunctive ultrasonography to screen for breast cancer in the Japan Strategic Anti-cancer Randomized Trial (J-START): A randomised controlled trial. Lancet.

[27] Honjo S., Ando J., Tsukioka T., Morikubo H., Ichimura M., Sunagawa M., Hasegawa T., Watanabe T., Kodama T., Tominaga K. (2007). Relative and Combined Performance of Mammography and Ultrasonography for Breast Cancer Screening in the General Population: A Pilot Study in Tochigi Prefecture, Japan. Jpn. J. Clin. Oncol..

[28] Zhou S.-C., Fan Y.-W., Zeng W., Ding J.-H., Chen M., Wang B.-H., Qiu Y.-F., Gao Y., Zhang X., Chang C. (2012). The first stage conclusion of breast cancer screening in Shanghai community: Missed and misdiagnostic cases analysis of mammography and ultrasonography. Shanghai Med. Imaging.

[29] Kang M., Zhao Y., Huang Y., Li J., Liu L., Li H. (2014). Accuracy and direct medical cost of different screening modalities for breast cancer among Chinese women. Zhonghua zhong liu za zhi.

[30] Shen S., Zhou Y., Xu Y., Zhang B., Duan X., Huang R., Li B., Shi Y., Shao Z., Liao H. (2015). A multi-centre randomised trial comparing ultrasound vs mammography for screening breast cancer in high-risk Chinese women. Br. J. Cancer.

[31] Dong H., Huang Y., Song F., Dai H., Liu P., Zhu Y., Wang P., Han J., Hao X., Chen K. (2018). Improved Performance of Adjunctive Ultrasonography After Mammography Screening for Breast Cancer Among Chinese Females. Clin. Breast Cancer.

[32] Xu J., Wang Q., Ma H.M., Xia J.H. (2013). Primary efficacy of physical examination combined with ultragraphy and complemented with mammography for breast cancer screening. Chin. J. Cancer Prev. Treat..

[33] Ma D., Wei N., Ni Q. (2014). The primary investigation on the incidence of breast cancer in the institution of Guiyang city. Chin. J. Clin. Oncol. Rehab..

[34] Lee E.H., Kim K.W., Kim Y.J., Shin D.-R., Park Y.M., Lim H.S., Park J.S., Kim H.-W., Kim Y.M., Jun J.K. (2016). Performance of Screening Mammography: A Report of the Alliance for Breast Cancer Screening in Korea. Korean J. Radiol..

[35] Suzuki A., Kuriyama S., Kawai M., Amari M., Takeda M., Ishida T., Ohnuki K., Nishino Y., Tsuji I., Shibuya D. (2008). Age-specific interval breast cancers in Japan: Estimation of the proper sensitivity of screening using a population-based cancer registry. Cancer Sci..

[36] Fan L., Strasser-Weippl K., Li J.-J., Louis J.S., Finkelstein D.M., Yu K.-D., Chen W.-Q., Shao Z.-M., Goss P.E. (2014). Breast cancer in China. Lancet Oncol..

[37] Najjar H., Easson A. (2010). Age at diagnosis of breast cancer in Arab nations. Int. J. Surg..

[38] Lee S.K., Society K.B.C., Kim S.W., Yu J.-H., Lee J.E., Kim J.Y., Woo J., Lee S., Kim E.-K., Moon H.-G. (2018). Is the high proportion of young age at breast cancer onset a unique feature of Asian breast cancer?. Breast Cancer Res. Treat..

[39] Fan L., Zheng Y., Yu K.-D., Liu G.-Y., Wu J., Lu J.-S., Shen K.-W., Shen Z.-Z., Shao Z.-M. (2009). Breast cancer in a transitional society over 18 years: Trends and present status in Shanghai, China. Breast Cancer Res. Treat..

[40] Rosenberg S., Newman L.A., Partridge A.H. (2015). Breast Cancer in Young Women. JAMA Oncol..

[41] Del Carmen M.G., Halpern E.F., Kopans D.B., Moy B., Moore R.H., Goss P.E., Hughes K.S. (2007). Mammographic Breast Density and Race. Am. J. Roentgenol..

[42] Chinese Anti-Cancer Association (2019). Chinese female breast cancer screening guideline. Chin. J. Clin. Oncol..

[43] Rebolj M., Assi V., Brentnall A., Parmar D., Duffy S.W. (2018). Addition of ultrasound to mammography in the case of dense breast tissue: Systematic review and meta-analysis. Br. J. Cancer.

[44] Lee J.M., Arao R.F., Sprague B.L., Kerlikowske K., Lehman C.D., Smith R.A., Henderson L.M., Rauscher G.H., Miglioretti D.L. (2019). Performance of Screening Ultrasonography as an Adjunct to Screening Mammography in Women Across the Spectrum of Breast Cancer Risk. JAMA Intern. Med..

[45] Lehman C.D., Arao R.F., Sprague B.L., Lee J.M., Buist D.S.M., Kerlikowske K., Henderson L.M., Onega T., Tosteson A.N.A., Rauscher G.H. (2017). National Performance Benchmarks for Modern Screening Digital Mammography: Update from the Breast Cancer Surveillance Consortium. Radiology.

[46] Nelson H.D., O’Meara E.S., Kerlikowske K., Balch S., Miglioretti D. (2016). Factors Associated with Rates of False-Positive and False-Negative Results from Digital Mammography Screening: An Analysis of Registry Data. Ann. Intern. Med..

[47] Weigel S., Heindel W., Heidrich J., Heidinger O., Hense H.W. (2016). Reduction of advanced breast cancer stages at subsequent participation in mammography screening. RöFo.

[48] Hofvind S., Geller B.M., Skelly J., Vacek P.M. (2012). Sensitivity and specificity of mammographic screening as practised in Vermont and Norway. Br. J. Radiol..

