# Supplementary material for: Is Ultrasound an Accurate Alternative for Mammography in Breast Cancer Screening in an Asian Population? A Meta-Analysis
Source: Diagnostics (Basel). 2020 Nov 21;10(11):985. doi: 10.3390/diagnostics10110985 (PMC7700617; doi:10.3390/diagnostics10110985)
Supplement: Supplementary file 1 [file diagnostics-10-00985-s001.pdf]

Table S1. Search strategy in MEDLINE/PubMed.

	Query
#1	"breast neoplasms"[MeSH Terms] OR "breast"[MeSH Terms] OR "breast*"[Title/Abstract] OR "breast cancer*"[Title/Abstract] OR "breast neoplam*"[Title/Abstract] OR "breast tumor*"[Title/Abstract] OR "breast carcinoma*"[Title/Abstract]
#2	"mass screening"[MeSH Terms] OR "mammography"[MeSH Terms] OR "ultrasonography, mammary"[MeSH Terms] OR "screen*"[Title/Abstract] OR "mammo*"[Title/Abstract] OR "ultrason*"[Title/Abstract]
#3	"asia"[MeSH Terms] OR "asian continental ancestry group"[MeSH Terms] OR "China"[Title/Abstract] OR "Japan"[Title/Abstract] OR "republic of korea"[Title/Abstract] OR "Mongolia"[Title/Abstract] OR "Singapore"[Title/Abstract] OR "Vienam"[Title/Abstract] OR "India"[Title/Abstract]
#4	"Sensitivity and Specificity"[MeSH Terms] OR "specificit*"[Title/Abstract] OR "sensitiv*"[Title/Abstract] OR "screening"[Title/Abstract] OR "accura*"[Title/Abstract] OR "reference value*"[Title/Abstract] OR "false positive"[Title/Abstract] OR "false negative"[Title/Abstract] OR "predictive value*"[Title/Abstract] OR "roc"[Title/Abstract] OR "likelyhood*"[Title/Abstract] OR "likelihood*"[Title/Abstract]
Final	#1 AND #2 AND #3 AND #4 Filters: Publication date from 2000/01/01
